# Male Sexual Hemicrania

**Published:** 1882-11

**Authors:** R. W. Conantin


### SELECTIONS.

### MALE SEXUAL HEMICRANIA.

The following case is submitted, not so much because of anything remarkable in the pathology or treatment, as because it apparently belongs to a rare and neglected species. I say "apparently," for faithful search of all the works accessible to me revealed not a hint of description or treatment.

The case, stated as briefly as possible, and omitting non-essentials, was as follows: A man 30 years of age, a student most of his life, complains of terrible sick headaches which have troubled him with varying intensity so far back as he can remember. The symptoms are those of an ordinary megrim, plus the following:

The attack is very regular in coming once a week, and if by any chance omitted, there are two the next week to make up, so that he has just about fifty-two annually. The immediate cause is usually late hours, or too hearty or unwholesome food the previous evening. Headache begins at nine or ten A. M., and ceases only after vomiting or after night's sleep. Semilateral and pretty regularly alternate. Pain chiefly above eye, but also over whole side of head and down neck. Mind either lively or dull, and likes company.

Toward noon comes on nausea with chilliness and shuddering, hands and feet clammy, and jerking of legs. Sometimes violent retching and vomit, which is greenish, bitter mucus. Always hard to vomit and requires double dose of emetic.

Tenesmus vesicæ throughout the day of the attack, but amount of urine normal. Next day urine normal, except in being very dark. Weak in small of back the first day, and sore the next.

With the tenesmus, erotism and erections. Emission generally occurs before attack, and its occurrence always signifies coming headache.

The patient has dark brown hair and eyes, is slight and a little below medium height. Broad, flat tongue, with prints of teeth; broad, short nose, full hips. Father and mother never have sick headaches, and very healthy. One sister has them same as himself, only worse. Rheumatism runs in the family, and he has had a little, but cannot recall any connection between it and the headaches, either as amelioration or aggravation.

He has several personal peculiarities. Has been much exposed to malaria, and nursed typhoid fever patients with entire immunity from either disease, and can remember no sickness save croup and measles. In short, his life-long headaches seem to have diverted all other morbific tendencies into that one channel.

Another peculiarity : He has unusual sexual activity ; inherited, as he thinks. Has masturbated, in youth, but has fought and finally subdued the habit. Has otherwise led a virtuous life and endeavored always to keep his thoughts pure. Nevertheless suffers greatly from ungratified longings.

These statements I had reason to believe unusually accurate and reliable, coming from a man of education and refinement, and an old personal friend. Says I to myself : Here's a fine chance for some hard study and brilliant results with high potencies. For I believe in the Skyward, test tube and microscope to the contrary notwithstanding. I was bound to do my prettiest, for this man had tried many of the best physicians, East and West, of both schools, and with little or no benefit.

I consulted all my authorities. Not a word to assist me. Of course here were "key notes" by the bushel ; in fact, a fatal superfluity. I notice that there are generally too many or none at all. But what I wanted was some assistance in discovering the essential peculiarity which differentiated this case of megrim from others ; in short, what was the true and ultimate cause. Of course such routine prescriptions as *nux*, *ipecac*, *bell.*, etc. were out of the question.

So I was obliged to fall back on myself, and evolve my own theories. These were : that the affection was a masked rheumatism, and hereditary. That it was masked malaria. That it was of sexual origin, etc. After much mental balancing, I adopted the theory that it was hereditary, and looked up *sulphur*, and of course easily found all the symptoms under that especially accommodating drug. So *sulph.* $^{200}$ was "exhibited." After a fair trial the "exhibition" was closed. Results, *Nil.*

That had been my pet theory, and it seemed shattered. So I went to work more vigorously than ever ; and Allen's unrivalled *omnium gatherum*, and many other books were thumbed over by the hour. The new plan of attack was arranged in accordance with the sexual theory, and the standard was *conium mac.* $^{200}$. A Waterloo. Still I would not give *conium* up, for I was sure it was the simillium, and the $3^{x}$ took a turn. No better.

My third best choice had been *agaricus mus.* This given in the $3^x$ stopped the headaches almost immediately. A few relapses have been due to failure to take the medicine, but even these have been quite slight, mere rumblings of the vanishing storm. Finding the $3^x$ act so well, some $12^x$ was prepared and given him to take once a week ; reserving the $3^x$ for any threatened attack.

The patient reports himself much improved also in physical condition every way. The sexual organs in particular are firmer and more vigorous, while at the same time the erotism is diminished very much.

The result of the treatment seems to justify the theory that this was a genuine case of hemicrania in the male due to suppressed sexual activity and continence.* Unfortunately, a very small proportion of our young men are threatened with disease from such a cause ; yet there are such men, all honor to them, and their sufferings are often quite as severe as those who suffer from an opposite reason, and they deserve all the relief which medicine can give. It is not enough to say that the consciousness of virtue should be its own reward, for there is a sting in the very thought that virtue should bring suffering only, where *discreet* sin might bring relief and health.

If the description of this case shall help my fellow-physicians in leading some sufferer along a path where there are few or no guide boards, it has not been written in vain.—*R. W. Conantin, N. Y. Med. Times.*

---

* And yet these headaches had been troubling the patient "*so far back as he could remember!*" Either Dr. C. is at fault in his etiology, or he has failed to take notice of the most remarkable feature in the case !—Eds.

